# COVID-19 Severity in Obesity: Leptin and Inflammatory Cytokine Interplay in the Link Between High Morbidity and Mortality

**DOI:** 10.3389/fimmu.2021.649359

**Published:** 2021-06-18

**Authors:** Radheshyam Maurya, Prince Sebastian, Madhulika Namdeo, Moodu Devender, Arieh Gertler

**Affiliations:** ^1^ Department of Animal Biology, School of Life Sciences, University of Hyderabad, Hyderabad, India; ^2^ Institute of Biochemistry, Food Science and Nutrition, The Hebrew University of Jerusalem, Rehovot, Israel

**Keywords:** COVID-19, leptin, obesity, inflammation, cytokine, mortality

## Abstract

Obesity is one of the foremost risk factors in coronavirus infection resulting in severe illness and mortality as the pandemic progresses. Obesity is a well-known predisposed chronic inflammatory condition. The dynamics of obesity and its impacts on immunity may change the disease severity of pneumonia, especially in acute respiratory distress syndrome, a primary cause of death from SARS-CoV-2 infection. The adipocytes of adipose tissue secret leptin in proportion to individuals’ body fat mass. An increase in circulating plasma leptin is a typical characteristic of obesity and correlates with a leptin-resistant state. Leptin is considered a pleiotropic molecule regulating appetite and immunity. In immunity, leptin functions as a cytokine and coordinates the host’s innate and adaptive responses by promoting the Th1 type of immune response. Leptin induced the proliferation and functions of antigen-presenting cells, monocytes, and T helper cells, subsequently influencing the pro-inflammatory cytokine secretion by these cells, such as TNF-α, IL-2, or IL-6. Leptin scarcity or resistance is linked with dysregulation of cytokine secretion leading to autoimmune disorders, inflammatory responses, and increased susceptibility towards infectious diseases. Therefore, leptin activity by leptin long-lasting super active antagonist’s dysregulation in patients with obesity might contribute to high mortality rates in these patients during SARS-CoV-2 infection. This review systematically discusses the interplay mechanism between leptin and inflammatory cytokines and their contribution to the fatal outcomes in COVID-19 patients with obesity.

## Introduction

Obesity is marked as redundant fat accumulation in the body. Obesity is considered an increased circulating fatty acid that is causing low-grade chronic inflammation due to macrophages’ chemoattraction and its expansion in the adipose tissue (1, 2). An individual with obesity presents with increased TNF-α cytokine, changed T-cell subset, and suppressed T-cell responses driving to a high possibilities of communicable diseases (3). Obesity has been shown to be associated with the production of dysregulated cytokines and augment the synthesis of acute-phase reactants, such as IL-6, IL-8, TNF-α, C-reactive protein (CRP), and monocyte chemotactic protein-1 (MCP-1) in patients with obesity and various animal models of obesity (4–7). Pre-conditioned chronic inflammation directly or indirectly impacts the immune response to infection in individuals with obesity (8). Several viruses, such as influenza A virus, Adenovirus Ad 36, cytomegalovirus (CMV), and HIV utilize adipocytes as cellular reservoirs for viral shedding (9). In the 2009 influenza pandemic, individuals with obesity exhibited more severe illness from the H1N1 virus and were reported as a nonpartisan threat for severe influenza infection and appended death (10). Many other reports have been confirmed the severity of disease and death from H1N1 influenza linked in a patient with obesity (11–13). Experimental studies have confirmed that obese mice show increased susceptibility to influenza, including increased lethality with H1N1 and H3N2 strains (14–17). A precise study of MERS-CoV cases reveals that diabetes and hypertension are equally predominant in 50% of the patients. In contrast, the chances of cardiac diseases and obesity are 30% and 16%, respectively. These conditions affect the pro-inflammatory cytokine responses, resulting in impairment of the host’s innate and humoral immune response among these patients (18). However, the critical mechanism for more inclusive COVID-19 severity and mortality in obesity remains unknown, despite how other viral infections can be providing some thought of COVID-19 illnesses and the severity of disease in an individual with obesity.

The connection between obesity and severity of viral infection, such as SARS and MERS, may be paramount in COVID-19 disease because of the genetic resemblance between the SARS-CoV-2 virus with SARS-CoV and MERS-CoV which is 80% and 50%, respectively (19). Patients with obesity in SARS-CoV-2 infection show severe clinical complications in the case of acute respiratory distress syndrome (ARDS), cardiac injury, thromboembolic disease, disseminated intravascular coagulation (DIC), and in severe chronic obstructive pulmonary disease (COPD) (20, 21). Acute lung injury (ALI) is another primary reason of severe illness and mortality among SARS-CoV-2–infected patients (22). Display of ARDS and ALI is cited as lung failure because of the hyper inflammatory response rather than the direct viral cytopathic injury (23, 24). Another important factor that could be contributing to the immune imbalance in COVID-19 patients with obesity is chronically higher adipokines, such as leptin. In influenza infection, leptin resistance is a major infection susceptibility factor in individuals with obesity by quashing the countenance of IFN-α, IFN-β, IFN-γ mRNAs, and memory T cells (25). It is thoroughly documented that leptin regulates the pro-inflammatory cytokines response to maintain the Th1/Th2 dichotomy. This review highlights the distinctive facts regarding the link between obesity and inflammation, specifically the interplay of leptin and inflammatory cytokine response leading severity and mortality among COVID-19 patients.

## Obesity Linked COVID-19 Pathogenesis in Human

A study reported from China showed COVID-19 patients with body mass index (BMI) oF 28.0 ± 2.5 display severe signs of symptoms compared with patients with BMI of 21.2 ± 2.7. It also showed that a cohort of patients with obesity has a high frequency of getting severe symptoms and admitted to the intensive care unit (26). Two independent studies of hospitalized COVID-19 patients in New York City reported that obesity is one of the utmost shared comorbidities along with hypertension and diabetes and is independently linked to worse in-hospital outcomes and higher death (27, 28). A meta-analysis of pooled data of 22 and 35 independent studies showed that persons with obesity increase the probabilities of staying more in an intensive care unit by 74% and likely to have unfavorable fallouts with a 48% upsurge in deaths of COVID-19 patients, respectively (29). As leptin levels are vastly associated with obesity, there is the yet unanswered question of whether leptin alone is responsible for a bad prognosis. Preliminary data from various studies revealed that younger adults with obesity are more vulnerable to a higher risk for intubation or mortality in COVID-19 infection (30, 31). The morbidity and mortality in a young patient with obesity are mainly because of the visceral adiposity, leading to multiple organ failure in severe COVID-19 illness (32). A retrospective cohort study of 65 admitted patients aged 18 to 40 years in Zhongnan Hospital, Wuhan showed that obesity is a vital parameter to determine the severity of COVID-19 infection among young patients (33). The computed tomography (CT) analysis of visceral fat revealed that severity was linked to the accumulation of ectopic fat in several visceral organs, such as the liver, epicardial, and kidney (33, 34). Other studies that included a cross-sectional analysis of fat distribution showed that it is critical to consider BMI as a risk factor apart from the overall obesity in COVID-19 patients. CT-based quantification of fat showed the increment in the visceral fat area (VFA) and upper abdominal perimeter augmented the COVID-19 severity in patients (35).

**Figure 1:**
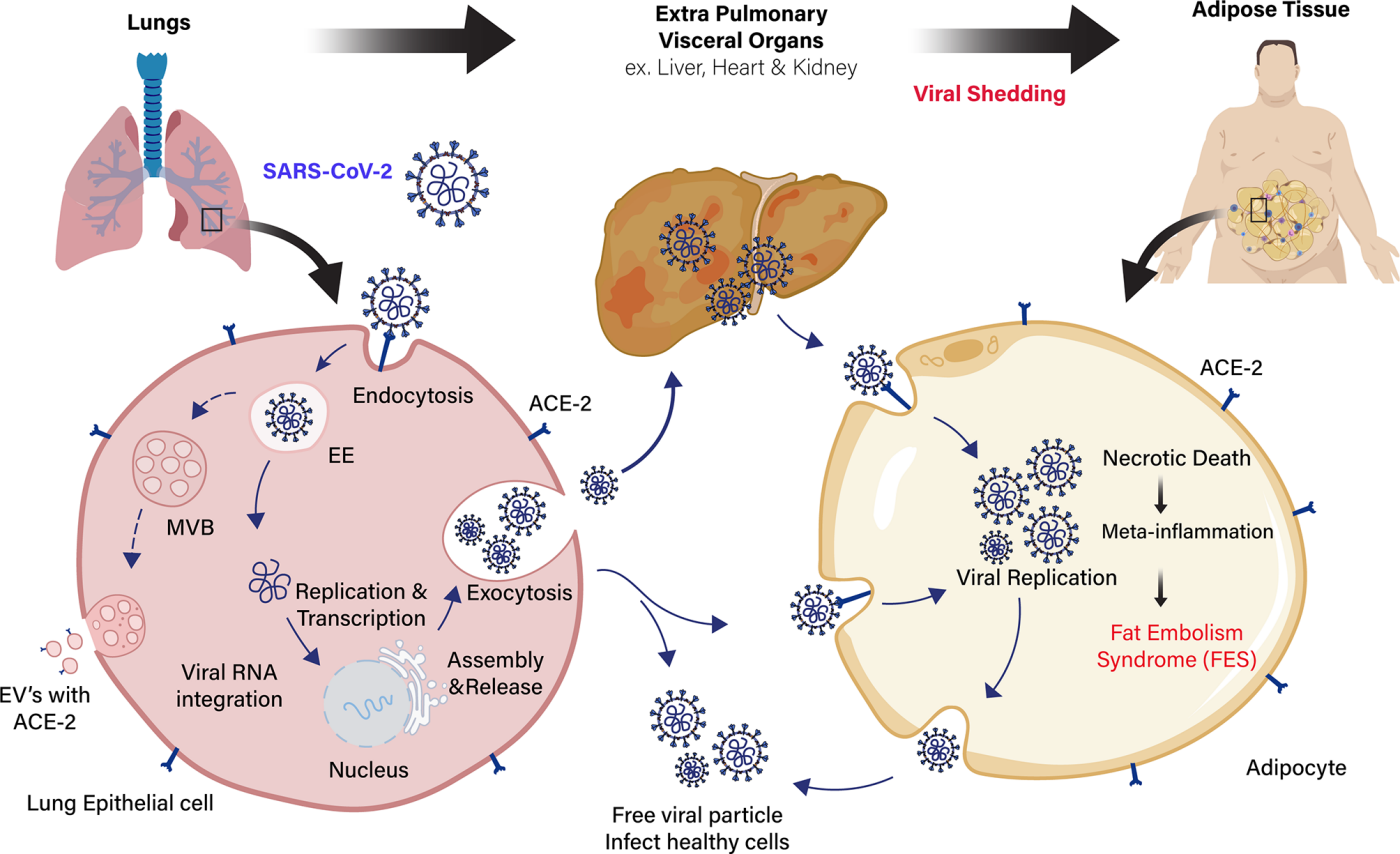
Schematic representation of extra pulmonary viral shedding and adipose tissue (AT) inflammation in COVID-19 patients with obesity. Angiotensin-converting enzyme 2 (ACE2) receptor in the lung epithelial cells recognizes the COVID-19 spike protein and the virus with receptor taken into the cells by receptor-mediated endocytosis. Then, viral RNA integrates into the host genome for viral replication and assembly. The free viral particles inside the host cell are released through exocytosis to infect healthy cells. Extracellular vesicles (EVs) containing viral proteins, and ACE2 receptors are also carried over to cells of extra pulmonary fatty visceral organs. The fatty adipose tissue in patients with obesity is a link to extra pulmonary viral shedding through the ACE2 expressing adipocytes. The adipocytes in the fatty AT of visceral organs (liver, heart, and kidney) act as a reservoir for viral replication and assembly. Apoptotic and necrotic death of the virus inside the adipocytes leads to meta-inflammation and subsequent fat embolism syndrome (FES) in COVID-19 patients with obesity.

The SARS-CoV-1 and SARS-CoV-2 virus entry into the adipocytes is mediated through the membrane protein angiotensin-converting enzyme 2 (ACE2) receptor (36, 37). It is vastly expressed in vascular tissues, such as the lungs and adipose tissue (38). ACE2 is expressed by a majority of cells in human at varying levels, including the intestinal enterocytes, liver, heart, and kidneys (39). The SARS-CoV-2 virus binds to the ACE2 receptors present in the host’s cell membrane. Subsequently, transmembrane serine protease (TMPRSS2) cleaves the viral capsid spike (S) protein and favoring the viral particle to infiltrates into host cells (40). The ACE2 expression in adipocytes appears to be stimulated by high-fat diets (41, 42). Adipocytes of individuals with obesity express a high level of ACE2 receptor, shedding more viral load than lean individuals, which exacerbate the susceptibility and severity of patients during COVID-19 infection (43–45). Several reports suggest that drugs used to treat patients with obesity complications (antihypertensives, statins, thiazolidinediones) could induce the overexpression of ACE2 receptor in adipocytes, leading to more viral uptake and severity of the disease (46–48) (**Figure 1**). Similarly, various extracellular vehicles (EVs) could also carry viral particles and ACE2 receptors from infected to healthy cells that could enhance COVID-19 susceptibility (43, 49). Fatty adipocytes in the visceral depots allow the SARS-CoV-2 entry through the ACE2 receptor (50), resulting in necrotic death of adipocytes in AT, which leads to a meta-inflammatory condition called fat embolism syndrome (FES). Strikingly, lung autopsy of an overweight patient with COVID-19 infection showed fat embolism macrophages infiltration in the AT (51, 52). The significant increase of inflammatory biomarkers, such as serum amyloid A, IL-6, and CRP in severe COVID-19 patients (53), are directly or indirectly linked to adipocytes with sub-clinical low-grade inflammation that further exacerbates COVID-19 severity in individuals with obesity (54, 55). Adipocytes is known to secrete leptin, which is an imperative mark of obesity and is directly proportional to body fat mass. Leptin involves distinct endocrine, paracrine, and autocrine signaling mechanism and cytokine-mediated inflammatory changes in the body (56). Therefore, leptin could play a significant role in developing severe COVID-19 infection in patients with obesity.

## Adipose Tissue Linked Inflammation

**Figure 2:**
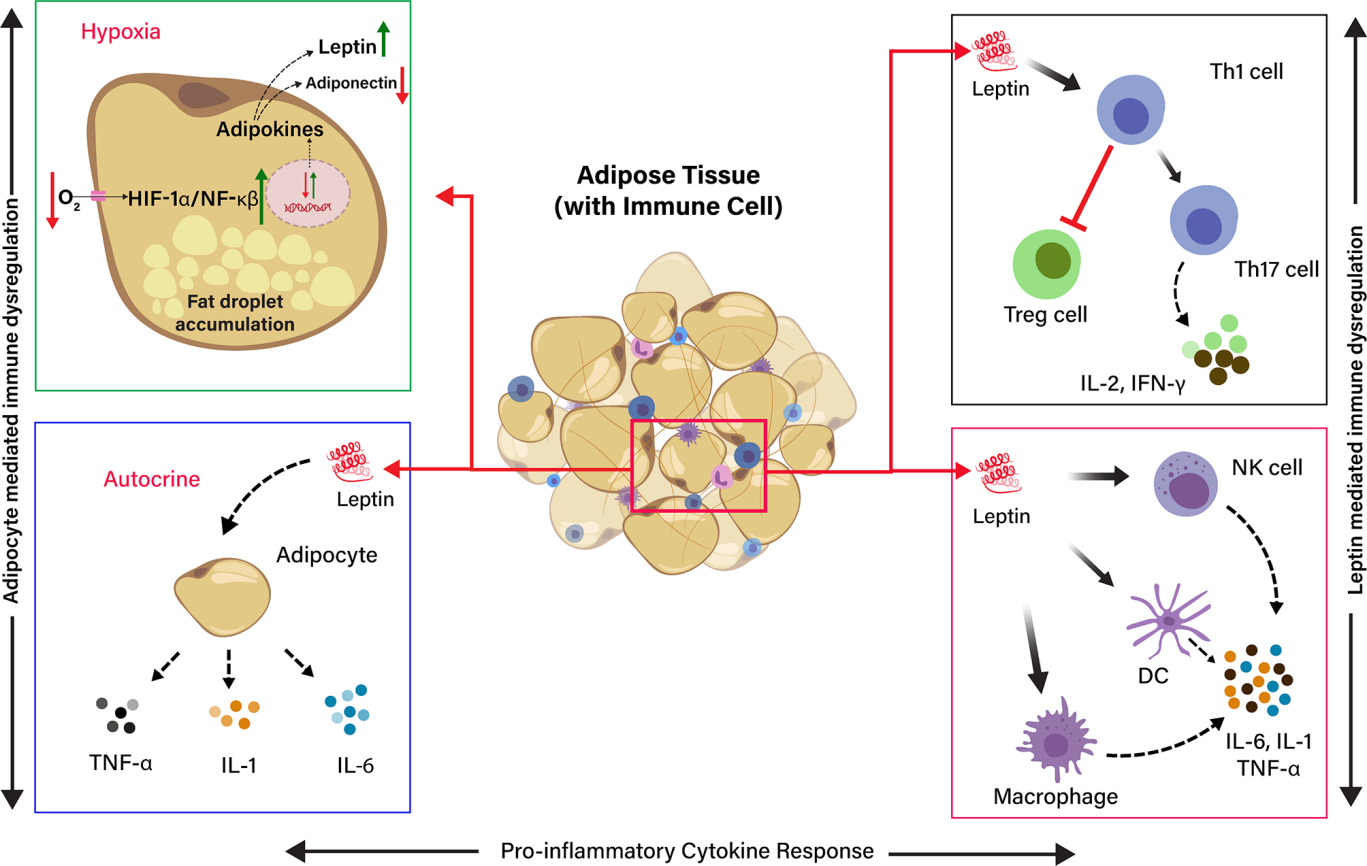
Schematic representation of the effects of Hypoxia and Leptin on pro-inflammatory cytokine response in adipose tissue (AT). The effect of low O_2_ tension on the production of adipokines in hypoxia condition. The adipocyte in the adipose tissue release leptin, which induces the release of pro-inflammatory cytokine by endocrine, paracrine, and autocrine in the AT. Leptin induces Th1 cells, and it inhibits T regulatory cells (Treg cells) and induces Th17 cells differentiation to produce IL-2 and IFN-γ. Leptin directly activates macrophages, dendritic cells (DC), and natural killer (NK) cells to produce pro-inflammatory cytokines, such as IL-6, IL-1, and TNF-α. Adipocyte-derived leptin induces the adipocytes to produce pro-inflammatory cytokines (IL-6, IL-1, and TNF-α) through autocrine signaling.

Adipose tissue is typically heeded as an inert tissue and functionally considered as an energy store house. It also deeds a central role in the immune and endocrine function that regulates inflammatory responses against infection. Visceral adiposity is associated with metabolic disorder and is recognized as a high-risk factor during SARS-CoV-2 infections (34). However, the exact mechanisms lying behind it are partly explained. Adipose tissue–linked inflammation is marked by the secretion of inflammatory cytokines, such as TNF-α, IL-6, and IL-1, from adipocytes and resident macrophages in adipose tissue (2). However, a typical feature of adipose tissue is adipocyte hypertrophy due to the accumulation of extended fat droplets. Adipocytes can reach a diameter of 150 to 200 microns in size, which limits the diffusion/exchange of gas (O_2_/CO_2_) in the adipose tissue. This produces a progressive local hypoxia by activating the hypoxia-inducible factor 1α (HIF-1α) and NF-κβ expression (57). The HIF-1α is a molecular sensor of hypoxia, and it transactivates the leptin gene expression by HIF-1α transcription-dependent activity (58). In addition to this, hundreds of immuno-inflammatory-reparative genes are also expressed, profoundly changing the adipocyte phenotype and functionality. Leptin is a product of a hypoxia-inducible gene, and hypoxia differentiates the preadipocytes into leptin secreting endocrine cells in an mTOR-dependent manner. Preadipocyte-derived leptin activates inflammatory cytokines by a specific local autocrine/paracrine signaling (57, 59) (**Figure 2**).

Adipocytes also secrete numerous adipocytokines, such as adiponectin, resistin, visfatin, and leptin. These molecules are believed to be linked with insulin resistance, inflammatory disorders, and obesity (60–62). Adiponectin plays a vital role in the secretion of anti-inflammatory cytokines, like IL-10 and IL-1 receptor antagonist (IL-1RA), by inducing the monocytes, macrophages, and dendritic cells (DCs). Adiponectin also plays a significant role in suppressing the interferon-γ (IFN-γ) secretion in LPS-stimulated macrophages (63, 64). Human resistin incites the secretion of the proinflammatory cytokines TNF-α, IL-1, IL-6, and IL-12 by several immune cell *via* NF-κB–dependent pathway (65, 66). It also upregulates the expression of vascular cell adhesion molecule-1 (VCAM-1), intercellular adhesion molecule-1 (ICAM-1), and CC-chemokine ligand-2(CCL-2) by human endothelial cells to release endothelin-1 (67). Serum amyloid A is another adipokines produced by adipocytes that enhances inflammatory cytokines production in individuals with obesity (68).

The primary function of leptin is to regulate appetite and energy (60). Nonetheless, leptin is also thought to be a proinflammatory cytokine, and it has a structural similarity with other inflammatory cytokines, such as IL-6, IL-12, and granulocyte colony-stimulating factor. Leptin promotes the migration of resident macrophages in the AT and stimulates their polarization toward a proinflammatory profile or a classically activated macrophage (M1) phenotype. It overturns the T helper cell phenotype by diminishing regulatory T-cells (Treg) activity and favoring Th17 polarization (69). Leptin modulates macrophage and T-cell function through the activation JAK-STAT pathway, leading to immune dysregulation and proinflammatory cytokine production (70, 71). Hyperleptinemia is another complication due to leptin resistance or leptin-impaired signaling. It interferes with the leptin receptor, contributing to the inflammatory response in obesity (72). Moreover, various other fatty tissue products have been characterized, such as TNF-α, IL-6, IL-1, CCL2, and plasminogen activator inhibitor type-1, which play a crucial role in immune dysregulation in individuals with obesity (68). Therefore, adipocyte interactions with immune cells (**Figure 2**) could be facilitating the disease severity in COVID-19 patients linked with obesity.

## Leptin Dysregulation Linked to Cytokine Storm in COVID-19 Infection

Leptin is a non-glycosylated 16-kDa protein encoded by the obese (ob) gene and mainly secreted by adipocytes to the circulation. Leptin acts as a pleiotropic protein, like hormone, as well as a cytokine. The circulatory leptin binds to the long form of the leptin receptor (Lep-R), class I receptor cytokine family mediates the phosphorylation and activation of STAT3 signaling pathway (71, 73). Lep-R is expressed in most of the immune cells, and leptin binds and triggers the immune response mainly through the JAK-STAT– and NF-kB–dependent pathway (69, 71, 74). Leptin functions in immune response have been confirmed in leptin-deficient (ob/ob) and leptin receptor-deficient (db/db) mice (75–77). The genetically leptin-deficient or leptin-resistant animals or humans develop profound obesity because of hormonal and immune system abnormalities (77–79). Leptin secretion in individuals with obesity is chronically higher than lean subjects. The higher level of pro-inflammatory leptin and lower anti-inflammatory adiponectin ratio could be contributing to the obesity-associated complication due to biased immune response (80). Leptin can tempt the stimulation and proliferation of various immune cells, including circulating monocytes, natural killer cells (NK) cells, and T helper cells by increasing the release of inflammatory cytokines, such as TNF−α, IL−1, and IL−6 (81–83). Leptin has structural homology with the long-chain helical family’s cytokines, such as IL-6, IL-11, IL-12 cytokine, and oncostatin M (84). Leptin has a shared signal-transducing peptide portion for the IL-6-related cytokines, such as IL-6, IL-11, leukocyte inhibitory factor (LIF), and granulocyte colony-stimulating factor (GCSF) (69, 85). Along with these, certain inflammatory and infectious stimuli are also enhancing the leptin expression, such as TNF-α, IL-1, IL-6, and lipopolysaccharide (LPS) (86). Therefore, the crucial question is whether or not the cytokine storm in COVID-19 patients with obesity is linked to leptin dysregulation.

Adipocytes engage in a cross-talk with various immune cells and could be directly involved in their activation and proliferation, resulting in the production of inflammatory cytokines (87, 88). Adipocytes add nearly one third of the IL-6 concentration to the circulation, along with macrophages that are recruited into the AT by leptin (89). IL-6 is the foremost cytokine in acute phase inflammation, an innate immune response elicited by infection. IL-6 also plays an indispensable role in improving acquired immunity against pathogens, including cytokine and chemokine expressions, stimulation of antibody production from B cells, macrophage regulation, and dendritic cell differentiation (90, 91). The macrophage deposition and its chronic activation cause the abnormal secretion of IL-6, TNF-α during SARS-CoV-2 infection in a patient with obesity (51, 92). An altered pro-inflammatory profile and hyperleptinemia lead to thrombotic risk in these patients that are linked to coagulation of blood, resulting in visceral organ failures (90, 93). In addition, leptin modulates the gene expression in cardiomyocytes *via* hypoxia-mediated HIF-1α factors, which is the leading cause of heart ischemia (94). In a recent cross-sectional study in 31 COVID-19–positive patients, interestingly, the serum leptin levels of ventilated patients were significantly higher compared with the control groups (95). Adipocyte dysfunction linked to leptin could be contributing to the expansion of ARDS in COVID-19 patients. Therefore, a strong possibility of neutralizing the circulatory leptin could control the hyper cytokine responses that are linked to obesity in COVID-19 patients.

## Interplay Between Leptin and IL-6 in COVID-19 Infection

**Figure 3:**
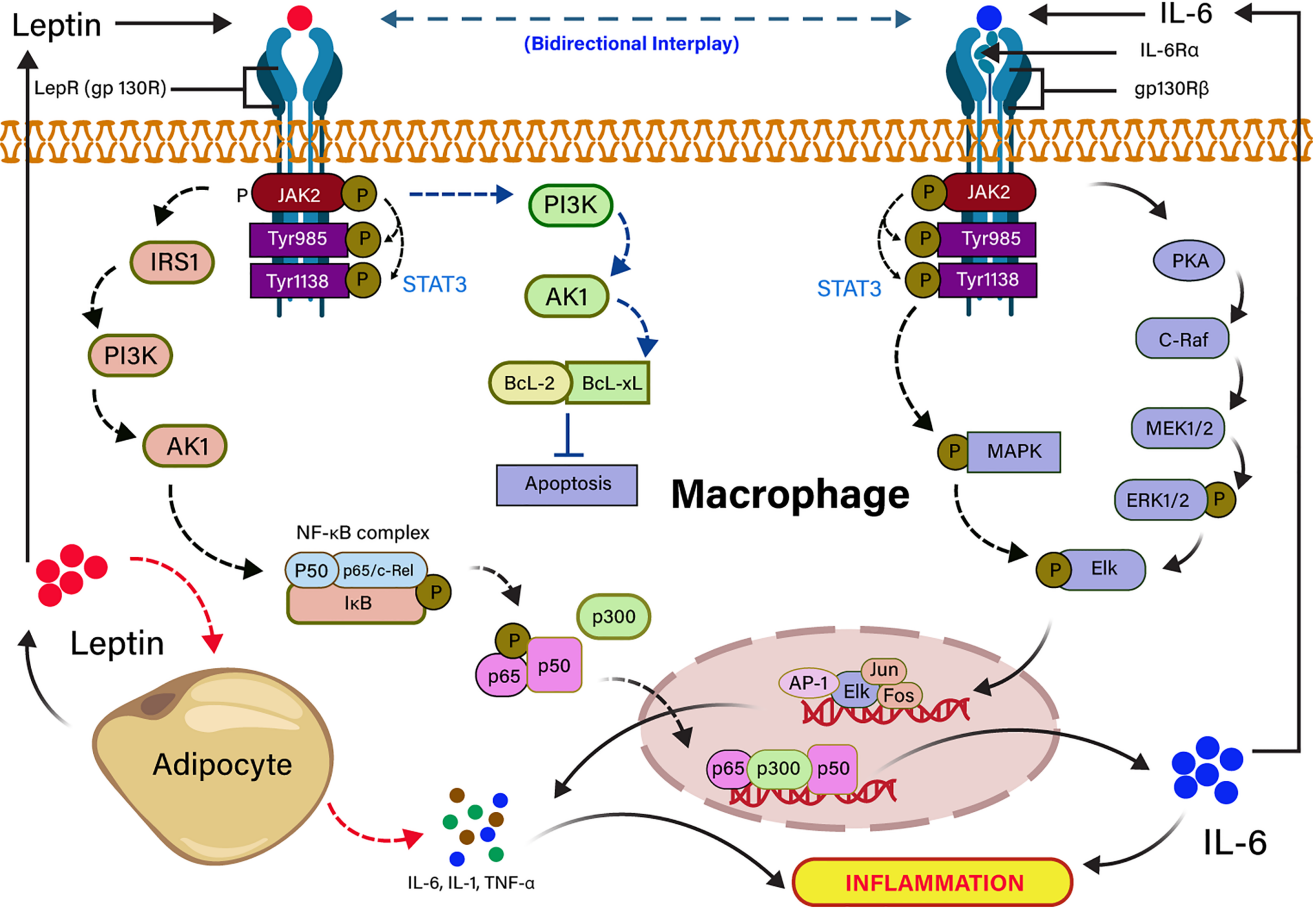
Signal transduction cascades of leptin and IL-6 interplay in inflammation. Leptin binding to the long-form leptin receptor (LRb or gp130R) results in the activation of the Janus kinase 2 (JAK2) signaling pathway by autophosphorylation, which subsequently phosphorylates tyrosine 985 and 1138 residues of the intracellular receptor tail. The phosphorylated Tyr 1138 activates the transcription factor STAT3. The activated JAK/STAT3 activates IRS1 and PI3K downstream signaling pathway, which recruits NF-kB complex (P50, P65/c-Rel, IkB), resulting in the transcription of IL-6 expressing genes in the nucleus. JAK/STAT3 directly activates PI3k inhibits apoptosis by the activation of anti-apoptotic factors (BCL-2, BCL-XL). Pleiotropic leptin and IL-6 follow similar signaling through gp130R and gp130Rβ, respectively. Leptin-induced IL-6 binding to the gp130Rβ results in the activation of JAK/STAT followed by the activation of PKA- and MAPK-mediated Elk phosphorylation. The phosphorylated Elk induces the transcription factors for TNF-α and IL-1. IL-6–mediated TNF-α and IL-1 induce the production of leptin from adipocytes.

Severe COVID-19 patients show an expressively higher serum IL-6 than those with mild infection (96). High level of IL-6 is also another key signature of severe SARS-CoV, MERS-CoV, and pandemic H1N1 influenza A viral infections (97–99) In individuals with obesity, adipocytes secrete pleiotropic leptin and pleiotropic adipocyte-specific IL-6 cytokine (91, 100). IL-6–mediated inflammation in obesity is source-specific, and the IL-6 secreted by adipocytes or infection of non-resident adipocyte cells, including adipose tissue macrophage (ATM) and T cells in a non-canonical mechanism (101). Visceral adipose tissue (VAT) produces higher inflammatory cytokines (TNF-α, IL-6, and IL-1β) in comparison to subcutaneous adipose tissue (102), suggesting that cytokine storm in COVID-19 infection could be independent of obesity. IL-6 is released from adipocytes, either binding to a soluble or membrane-bound receptor, and forms an IL-6 ligand/receptor complex. IL-6 is also associated with a homodimer of glycoprotein 130 for downstream signaling in adipocytes (103, 104). Leptin and IL-6 act in agreement with partly sharing a common downstream target mediated by phosphorylation of STAT3 in ventromedial hypothalamic leptin signaling (105, 106). Furthermore, the structural and functional similarity of leptin and IL-6 receptor-mediated signaling *in-vitro* showed that both are activating the JAK-STAT pathway (82, 107). Leptin induces IL-6 expression through the binding to the leptin receptor (LEPR) and activates JAK2/STAT-3–dependent phosphorylation of IRS-1, PI3K, Akt signaling pathway (72). The binding of p65 and p50 transcription factor to the NF-κB element enhances the recruitment of p300 to the IL-6 promoter, resulting in up-regulation of IL-6 expression (91, 104). Conversely, IL-6 upregulates the expression of TNF-α and IL-1 through JAK/STAT-dependent phosphorylation of PKA signaling pathway (108). The dose-dependent effects of TNF-α and IL-1 cytokines on leptin gene overexpression revealed that their anorectic effects might be arbitrated in bit by expression of the leptin gene in the adipocyte (82, 109). Leptin also inhibits the apoptosis through the activation of PI3K-dependent Bcl-2 and Bcl-xL signaling pathways (110). The high leptin concentration in serum is correlated with incremental death in patients with ARDS caused by an infectious agent (111). It has also been stated that leptin induces an inflammatory phenotype in murine alveolar macrophages, hinting that a higher concentration of leptin in obesity might be potentiating the incidence of pulmonary inflammation in COVID-19 infection (112). The dysregulation of leptin in COVID-19 patients could be linked to IL-6 cytokine signaling because it shares the IL-6 receptors. The mechanism of leptin and IL-6 signaling pathways are summarized in **Figure 3**.

Another interesting study showed that leptin and IL-6 induce anorexia by acting on the lateral parabrachial nucleus (lPBN) in the brain, suggesting that targeting leptin might be controlling the IL-6 signaling in individuals with obesity (113). The elevated level of IL-6 in serum due to increased production of adipokines leads to induce low-grade chronic inflammation (114). Although high IL-6 in COVID-19 infection has a prominent role in inflammation linked mortality, recent studies showed that colchicine treatment decreased the concentrations of multiple inflammatory molecules, including IL-6, CRP, and resistin in patient with obesity (115). Tocilizumab is used to treat cytokine release syndrome (CRS) in patients with cancer treated with chimeric antigen receptor-modified (CAR) T cells. It inhibits the expression of IL-6 through the blocking of the IL-6, receptor resulting in inhibitions of proinflammatory cytokine, such as TNF-α, IL-1, and IFNγ (116, 117). In an early pandemic, it was reported that IL-6 plays a significant role in the cytokine storm in COVID-19 patients, opening new hopes to treat the cytokine storm by blocking the IL-6 cytokine receptor. However, later on, multicenter studies reported that there is no significant improvement after blocking the IL-6 receptor in these patients (118). Subsequently, IL-6 is not the only cytokine, several other cytokines, such as TNF-α, IL-1, and IL-8, have also been elevated in CRS and are equally important in acute inflammatory reactions that contribute to tissue injury. TNF-α and IL-1 blockade might be another probable therapeutic approach to restrained organ damage in patients with SARS-CoV-2 infection (119, 120). In vitro studies showed that adiponectin also inhibits IL-6 expression by murine pulmonary endothelial cells and reduces lung inflammation in mice during ARDS induced by bacterial liposaccharides (121). However, data if adipokines can regulate pulmonary inflammation in COVID-19 infection are lacking so far. Despite the fact that blocking the specific cytokines could be a strategy to treat COVID-19 patients, there is a possibility it may lead to severe outcomes. Therefore, the bidirectional interplay of cytoadipokines might play a role in non-specific immune response, such as cytokine storm in obesity-related mortality in severe COVID-19 patients.

## Prospective of LeptinTreatment in COVID-19 Infection

Because obesity is strongly correlated with poor prognosis in COVID-19 infection and because hyperleptinemia is characteristic of obesity, we suggest that a possibility lowering leptin activity may improve such bad prognosis. This can be achieved by blocking leptin action using leptin receptor antagonists (L39A/D40A/F41A mutants of human/mouse/rat/ovine leptins) (122) developed by our groups. The antagonistic activity of those mutants was up to 60-fold elevated by additional mutation (D23L), resulting in a respective super active D23L/L39A/D40A/F41A mutants (123). In view of the potential pharmaceutical uses of recombinant leptin mutants acting as antagonists, the basic question of how the biopotency of recombinant proteins can be elevated *in vivo* needs to be investigated. The capability of a hormone or hormone antagonists to elicit a biological effect *in vivo* depends not only on the affinity for its receptor but also on its clearance from the circulation. The measured half-life in circulation of leptin is or leptin antagonist only ~ 60 min (123, 124). As kidney-mediated clearance is mostly reliant on molecular mass and proteins greater than 70 to 80 kDa are cleared at a remarkably slower rate, increasing protein size will prolong its *in vivo* half-life. This can be attained by increasing the size of the hormone without affecting its activity, *via* attachment of polyethylene glycol (PEG) molecule. Therefore, to extend the biological activity of those mutants in animals by prolonging their *in vivo* half-life in circulation, they were mono-pegylated at their amino-terminus by 20-kDa PEG (123, 124) and termed PEG-SMLA (mouse) or PEG-SHLA (human) or PEG-SRLA (rat). Although the molecular weight of those analogs is ~ 36 kDa, due to the very large hydrodynamic volume of PEG, they behave like 220-kDa proteins, and their half-life in mice circulation was extended to ~ 14 to 15 h. The *in vivo* effect of those mono-pegylated analogs was well-documented in Prof. Gertler’s and in other laboratories (125–129). By applying different amounts of leptin antagonists, it is possible to control the leptin activity in serum.

## Conclusion

The interplays between leptin and inflammatory cytokine response that regulate the immune-metabolic feats of leptin on immune cells are required to explore the insight of their relation. The past two decades of studies about leptin reveals that adipose tissues play a crucial role in regulating metabolism and immune cell function. Adipose tissue inflammation is marked by infiltration of immune cells, such as lymphocytes, macrophages, dendritic cells, and neutrophils into it. Leptin expands the monocyte, macrophage, and T-cell proliferation by enhancing inflammatory cytokines secretion. Leptin also plays a major role in the anorexia and cachexia condition of inflammatory diseases caused by pathogens, collagen vascular disease, and even cancer. However, chronic inflammation, either from autoimmune or infectious diseases, or impaired leptin response to CNS (hypothalamus)/leptin resistance, induce resistance to the weight control, which is a leading cause of obesity and anorexia. The absence of leptin triggers the immune defects that eventually translate into exaggerated death due to infections. Therefore, high circulating leptin might involve dysregulation of proinflammatory cytokine in obesity, which is the leading cause of high morbidity and mortality in patients with SARS-CoV-2 infection. Individuals with obesity suggest that metabolic consequences of obesity compromise host antiviral defenses, leading to critical outcomes from infection. This could be one of the reasons why the virus has taken its toll to damaged individuals with obesity more aggressively. Therefore, more studies in the mechanism of leptin are warranted to investigate the immune response in COVID-19 patients with obesity.

## Author Contributions

RM and AG conceived and designed the study. PS, MN, and MD have done literature collection and wrote the initial version of the draft. RM and PS have prepared the figures and finalized the manuscript. All authors contributed to the article and approved the submitted version. RM is the guarantor and takes all responsibility for the integrity and accuracy of the data.

## Conflict of Interest

The authors declare that the research was conducted in the absence of any commercial or financial relationships that could be construed as a potential conflict of interest.
